# Critical illness induces nutrient-independent adipogenesis and accumulation of alternatively activated tissue macrophages

**DOI:** 10.1186/cc12378

**Published:** 2013-03-19

**Authors:** M Marques, S Vander Perre, A Aertgeerts, S Derde, F Guiza, M Casaer, G Hermans, G Van den Berghe, L Langouche

**Affiliations:** 1 KU Leuven, Belgium

## Introduction

In artificially fed critically ill patients, adipose tissue reveals an increased number of small adipocytes and accumulation of M2-type macrophages [1]. We hypothesized that nutrient-independent factors of critical illness explain these findings, and also that M2-macrophage accumulation during critical illness may not be limited to adipose tissue.

## Methods

We performed a randomized investigation in a septic mouse model of critical illness and a study of ICU patient biopsies. In the critically ill mouse, we compared the effect of parenteral nutrition (*n *= 13) with fasting (*n *= 11) on body composition, adipocyte cell size, and macrophage accumulation in adipose tissue, liver and lung. Fed healthy control mice (*n *= 11) were studied for comparison. *In vivo *adipose tissue was harvested after 1 week of illness from human patients (*n *= 40) who participated in a RCT on early parenteral nutrition versus tolerating nutrient restriction [2], adipose tissue morphology was characterized and compared with healthy controls (*n *= 13).

## Results

Irrespective of nutritional intake, critically ill mice lost body weight, total fat and fat-free mass. Part of the fat loss was explained by reduced ectopic fat accumulation. Adipocyte cell number and the adipogenic markers peroxisome proliferator-activated receptor γ and CCAT/enhancer binding-protein β increased with illness, again irrespective of nutritional intake. Macrophage accumulation with predominant M2-phenotype was observed in adipose tissue, liver and lungs of critically ill mice, further accentuated by fasting in visceral tissues. Macrophage M2-markers correlated with chemoattractant factor expression in all studied tissues. In human subcutaneous adipose tissue biopsies of critically ill patients, increased adipogenic markers and M2 macrophage accumulation were present irrespective of nutritional intake.

## Conclusion

Adipogenesis and accumulation of M2-macrophages are hallmarks of critical illness, irrespective of nutritional management in humans and mice. Critical illness evokes macrophage polarization to the M2-state not only in adipose tissue but also in liver and lungs, which is further accentuated by fasting.
